# *In vitro* and *in vivo* Phase Changes of the Mouse Circadian Clock by Oxidative Stress

**DOI:** 10.5334/jcr.136

**Published:** 2016-04-26

**Authors:** Yu Tahara, Aya Yokota, Takuya Shiraishi, Shunya Yamada, Atsushi Haraguchi, Ayako Shinozaki, Shigenobu Shibata

**Affiliations:** Laboratory of Physiology and Pharmacology, School of Advanced Science and Engineering, Waseda University, Tokyo, Japan; Waseda Institute for Advanced Study, Waseda University, Tokyo, Japan

**Keywords:** *Period2*, liver, kidney, *in vivo* imaging, hydrogen peroxide

## Abstract

Mammalian circadian rhythms are governed by an endogenous circadian clock system, including the molecular clock works in each cell and tissue. Adaptation of the circadian clock to different environmental stimuli such as light, food, and stress is essential for homeostasis maintenance. However, the influence of oxidative stress on the circadian clock phase is not fully understood *in vitro* and *in vivo*. Here, we examined the effects of hydrogen peroxide (H_2_O_2_)-induced oxidative stress on the PERIOD2::LUCIFERASE bioluminescence rhythm in mouse embryonic fibroblasts *in vitro* and in mouse peripheral tissues *in vivo*. The circadian clock phase changed with the dose of H_2_O_2_ and time of day *in vitro*; similar phase changes were observed *in vivo* in the circadian clocks of the peripheral tissues. In addition, mice treated with hemin-induced oxidative stress also showed phase changes of peripheral clocks, similarly as H_2_O_2_ treatment. Thus, oxidative stress can entrain circadian clock systems.

## Introduction

The circadian clock system in mammals plays important roles in maintaining physiology with day-night fluctuations. The central clock, located in the hypothalamus, organizes peripheral clocks in the other peripheral tissues through endocrine and nervous pathways [1]. Environmental stimuli such as light or food are essential for maintaining the clock phase to an appropriate time, because the potential period of internal clock oscillation is not 24 h (it is about 24 h) [2,3]. In addition to light and food, we recently found that physiological stress (e.g., restraint stress) has strong ability to synchronize the peripheral clock system *in vivo*; stress-induced sympathetic activation and glucocorticoid release are included in this stress-induced entrainment [4]. On the other hand, restraint stress also increases oxidative stress in the peripheral tissues [5,6]; however, the ability of oxidative stress to entrain the circadian clock system has not yet been elucidated *in vivo*.

Oxidative stress is seen as an imbalance of reactive oxygen species (ROS) such as O_2_– (superoxide radical), OH (hydroxyl radical), and H_2_O_2_ (hydrogen peroxide). Disruption of ROS maintenance causes many diseases and accelerates aging; therefore, anti-oxidative reactions need to be activated in order to decrease the ROS levels and to maintain homeostasis [7,8]. Interestingly, this response to ROS signaling has a day-night difference, because many anti-oxidative genes such as nuclear factor erythroid-derived 2-like 2 (*Nrf2*), a key anti-oxidant regulator, are under the regulation of circadian clock genes [9,10,11,12]. On the other hand, it is still unclear whether ROS activation could entrain the circadian clocks or cause phase changes. Thus, in the current study, we tried to determine the effect of H_2_O_2_ on the phase of PER2::LUC oscillation *in vitro* and *in vivo*, and the effect of stimulation timing during a day.

## Materials and methods

### In vitro PER2::LUC monitoring and H_2_O_2_ treatment

Bioluminescence oscillation of PER2::LUC mouse embryonic fibroblasts (MEF) cells was monitored by LumiCycle luminometer (Actimetrics, Wilmette, IL, USA) as previously described [13]. Briefly, after 2-h treatment with dexamethasone (Dex, 100 nM) to synchronize the circadian rhythm, the medium was changed to Dulbecco’s modified Eagle’s medium (DMEM) containing D-luciferin and 10% fetal bovine serum in order to monitor bioluminescence. Drug treatment was undertaken for 30 min at different timings, and the previous medium was returned to the dish after drug treatment, as previously described [13]. H_2_O_2_ (0.01–2 mM in water) was used in this study. The third peak of PER2::LUC was analyzed for phase changes in each group. Number of cells were analyzed by automated cell counter (Bio-rad, CA, USA) 4 days after starting bioluminescence monitoring.

### Animals

Heterozygous PER2::LUC knock-in mice [14] on the ICR background were used in this study. The mice (age, 2–6 months) were maintained on a 12:12-light/dark cycle (with lights on at 08:00 h) at room temperature (23°C ± 0.5°C) and were provided a standard moderate fat (MF) diet (Oriental Yeast Co., Ltd., Tokyo, Japan) and water *ad libitum*. The procedures conformed to the ‘‘Fundamental Guidelines for Proper Conduct of Animal Experiment and Related Activities in Academic Research Institutions’’ (published by the Ministry of Education, Culture, Sports, Science and Technology, Japan) and were approved by the Committee for Animal Experimentation of the School of Science and Engineering at the Waseda University (permission #2013-A061).

### In vivo PER2::LUC monitoring

Bioluminescence oscillation in peripheral tissues was monitored as previously described [15]. Images were acquired using an *in vivo* imaging system (Perkin Elmer, Waltham, MA, USA) with a 1-min exposure time from the dorsal- and ventral-up positions at 8 and 10 min after luciferin injection, respectively. Images were obtained 6 times a day at 4-h intervals (ZT7, 11, 15, 19, 23, and 3) using the same mouse. In each point, mice were anesthetized by isoflurane (Wako, Osaka, Japan), and returned to their home cage after imaging procedure. The average photon/s value for the 6 time points on each day was designated as 100%, and the bioluminescence rhythm for the entire day was expressed as a percentage of each set of the 6 time points for individual organs. The peak phase, amplitude, and rhythmicity of normalized data were determined using the single cosinor procedure program (Acro.exe version 3.5 by Dr. Refinetti). H_2_O_2_ (0.1–0.5 M in phosphate-buffered saline [PBS], 10 ml/kg of mouse body weight) or hemin (30 or 50 mg/kg, PBS for vehicle) was treated intraperitoneally to mice at ZT4 in this study.

### Locomotor activity monitoring

General locomotor activity was recorded with an infrared radiation sensor (F5B; Omron). Double-plotted actograms of locomotor activity are shown with 6 min epochs.

### Statistical analysis

Data were analyzed using GraphPad Prism (version 6.03; GraphPad software, San Diego, CA, USA). Equal variance and normal distribution tests were performed to select the appropriate statistical approach. Parametric or non-parametric analyses were conducted using a one-way or two-way ANOVA with Tukey, Sidak, Dunnett, or Dunn tests for post-hoc analysis. A p value of <0.05 was considered statistically significant.

## Results

### H_2_O_2_ – induced circadian clock phase shift in PER2::LUC MEF cells

**Figure 1:**
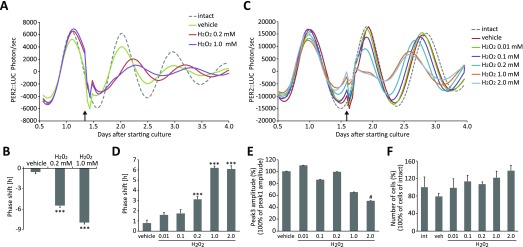
H_2_O_2_ treatment-induced dose-dependent phase-changes in the PER2::LUC rhythm in MEF cells. (A, C) Representative detrended data of PER2::LUC bioluminescence from MEF cells treated with water as a vehicle or 0.01-2.0 mM H_2_O_2_. Treatment was performed at Circadian Time (CT; CT0 indicates the trough of bioluminescence and CT12 indicates the peak) 18 (A) and CT4 (C) after the first peak of the PER2::LUC rhythm (indicated by the arrow). (B, D) Phase changes of the third peak in each treatment group, compared with the peak of intact. Phase delay (B) was seen in the experiment (A), and phase advance (D) was seen in the experiment (C). (E) Amplitude of the third peak was compared by bioluminescence (photon/sec). (F) Number of cells were counted after 4 days of luminescent monitoring. All values are mean ± SEM (n = 7–8 per group for A, n = 4 per group for C). ***p < 0.001 (vs. vehicle) by Dunnett test, #p < 0.05 (vs. vehicle) by Dunn test. int (intact); veh (vehicle).

**Figure 2:**
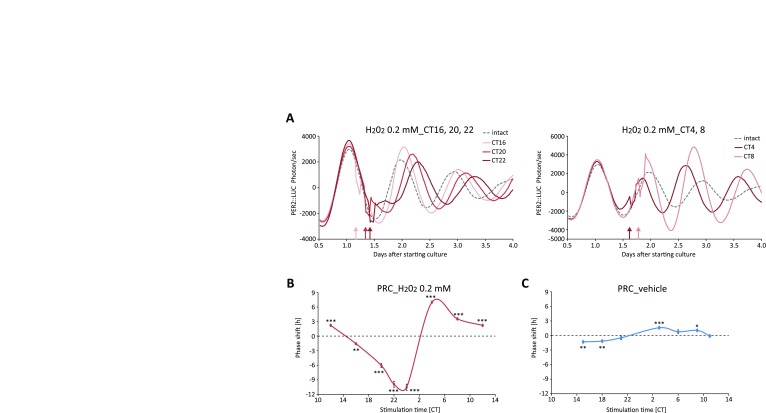
Phase response curve (PRC) of H_2_O_2_-induced PER2::LUC phase change in MEF cells. (A) Representative detrended data of PER2::LUC bioluminescence from MEF cells treated with 0.2 mM H_2_O_2_ at Circadian Time (CT; CT0 indicates the trough of bioluminescence and CT12 indicates the peal) 16, 20, and 22 (left panel), and CT4 and 8 (right panel). Arrows indicate the stimulation time points in each group. (B, C) PRC of PER2::LUC phase shift by 0.2 mM H_2_O_2_ (left panel) or vehicle (water, right panel). In the PRC, the peak change values of the third peak of bioluminescence of the intact and treated groups are indicated. All values are mean ± SEM (n = 4 for CT16, 20, 22, 28, and 32 in H_2_O_2_, n = 8 for CT12 and 24 in H_2_O_2_, and n = 4 for vehicle). *p < 0.05, **p < 0.01, ***p < 0.001 (vs. intact) by Sidak test.

The bioluminescence rhythms of the cells were monitored for 5 days, and the vehicle or H_2_O_2_ were added to the medium for 30 min at Circadian Time (CT; CT0 indicates the trough of bioluminescence and CT12 indicates the peak) 18 or CT4 after the first peak of the PER2::LUC rhythm (Fig. 1). We identified that H_2_O_2_ treatment at the time point when the slope of bioluminescence rhythm decreases caused phase delay of the third peak of the PER2::LUC rhythm in a dose-dependent manner, when compared to the vehicle-treated group. In addition, H_2_O_2_ treatment at the opposite time point (CT4) caused phase advance of the PER2::LUC rhythm in a dose-dependent manner without reduction of cell number in each group, when compared to the vehicle-treated group. H_2_O_2_ treatment (1 or 2 mM) decreased amplitude of PER2::LUC rhythms in this experiment. To further understand this phenomenon, a phase response curve (PRC) [13,16] was obtained by H_2_O_2_ treatment at different timings (Fig. 2). We found that H_2_O_2_ could strongly advance or delay the phases of PER2::LUC, relative to the vehicle-treated group. Phase delay occurred when H_2_O_2_ was treated during CT12-24 (the decreasing slope of PER2::LUC). On the other hand, phase advancement occurred during CT0-12 (the increasing slope of PER2::LUC). Thus, the oxidative stress induced by H_2_O_2_ treatment produced strong phase shifts of the circadian clocks *in vitro*, with time-of-day dependency.

### H_2_O_2_ – or hemin-induced phase shifts of the peripheral PER2::LUC rhythm in vivo

**Figure 3:**
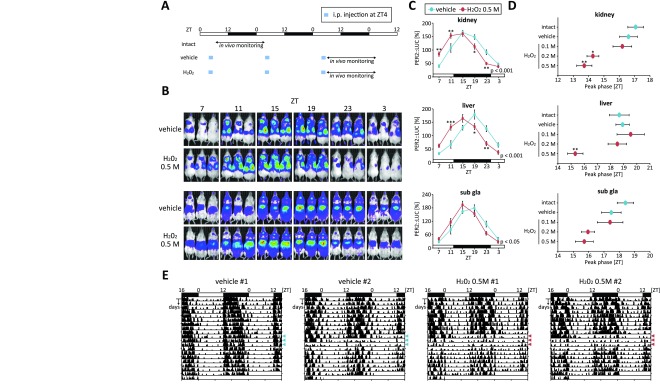
Phase changes in the PER2::LUC rhythm by H_2_O_2_ treatment *in vivo*. (A) Experimental schedule. PER2::LUC mice were intraperitoneally (i.p.) injected with the vehicle (PBS) or H_2_O_2_ (0.1–0.5 M) (10 ml/kg of mouse body weight) at ZT4 for 3 consecutive days. Then, the PER2::LUC rhythm of peripheral tissues was detected by an *in vivo* imaging system. (B) Representative images of *in vivo* PER2::LUC bioluminescence in the kidney (upper panels) and in the liver and submandibular gland (sub gla) (lower panels). (C) Normalized PER2::LUC oscillation in each tissue in vehicle and H_2_O_2_ (0.5 M) groups. P value on the lower right side of each graph indicates the results of two-way ANOVA between the vehicle and H_2_O_2_ groups. *p < 0.05, **p < 0.01, ***p < 0.001 (vs. intact) by Sidak test. (D) Peak phases of peripheral PER2::LUC oscillation in each group. All values are mean ± SEM (n = 6 in the intact group, n = 5 in the PBS and 0.25% 0.5 M H_2_O_2_ groups, n = 3 in 0.1 M H_2_O_2_). *p < 0.05, **p < 0.01 (vs. vehicle) by Dunnett test. (E) Representative locomotor activity data in vehicle or H_2_O_2_ (0.5 M) treatment. Injection was performed at ZT4 for consecutive 3 days (indicated by arrow heads).

**Figure 4:**
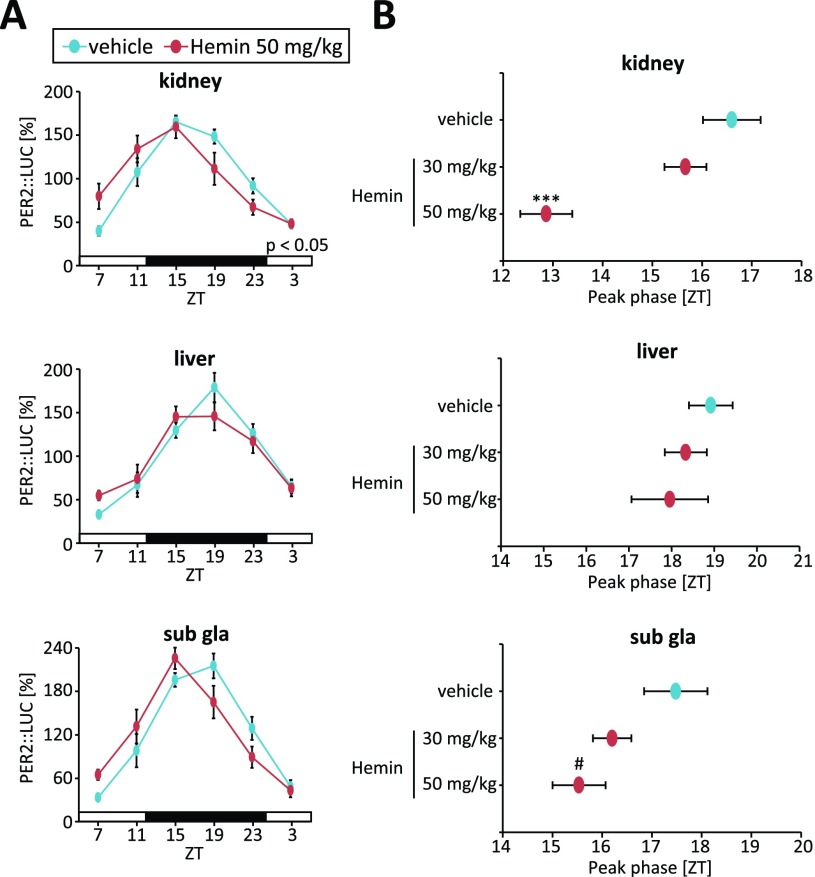
Phase changes in the PER2::LUC rhythm by hemin treatment *in vivo*. Experimental schedule was the same as Fig 3. Hemin (30 or 50 mg/kg, PBS for vehicle) was treated at ZT4 for 3 consecutive days, and then peripheral PER2::LUC rhythm was measured. (A) Normalized PER2::LUC oscillation in each tissue in vehicle and hemin (50 mg/kg) groups. P value on the lower right side of graph indicates the results of two-way ANOVA between the vehicle and hemin groups. (B) Peak phases of peripheral PER2::LUC oscillation in each group. All values are mean ± SEM (n = 5 in the PBS group and n = 6 in both hemin groups). ***p < 0.001 (vs. vehicle) by Dunnett test, #p < 0.05 (vs. vehicle) by Sidak test.

The effect of oxidative stress on the circadian clocks of mouse peripheral tissues was examined using an *in vivo* bioluminescence monitoring method [4,13,15]. A paradigm used for the current experiment was similar to our recent published method, which was used to study the effect of acute restraint stress on the phase of peripheral clocks [4] (Fig. 3). Intraperitoneal injection of H_2_O_2_ at Zeitgeber time (ZT) 4 for 3 consecutive days caused phase advancement in the PER2::LUC rhythm of peripheral tissues, strongly seen in the kidney and liver, and slightly seen in the submandibular gland. Additionally, these phase shift values showed a dose-dependent manner. The locomotor activities were reduced during night after treatment of H_2_O_2_, but the reduction was diminished after several days of H_2_O_2_ injection without no phase changes of behavioral rhythm. In addition, we confirmed this oxidative stress-induced phase changes by using another oxidative stress inducer hemin [17,18]. Injection of hemin at ZT4 also caused similar phase advance of peripheral PER2::LUC rhythms (Fig. 4). Thus, the oxidative stress could be an entrainable factor for the circadian clock systems not only *in vitro*, but also *in vivo*.

## Discussion

In this study, we found that the oxidative stress induced by H_2_O_2_ administration caused clear phase shifts of the PER2::LUC rhythm in the MEF cells *in vitro* and in the peripheral tissues *in vivo*, and that this phenomenon *in vitro* depended on the dose and the time of day.

Phase shift of the circadian clock by oxidative stress might include many signaling pathways. Oxidative stress activates Ca^2+^/CaMKII, heat shock stress-responsive HSF1, NF-κB, AMPK, or HIF [7], and the molecular clocks that interact with these pathways have already been reported. It is well understood that the Ca^2+^/CaMKII signaling is involved in light-dependent entrainment [16,19,20,21,22]. Tamaru et al. [23] reported that the ROS-induced phosphorylation of HSF1 and BMAL1 through CK2 caused transcription of *Per2* gene *in vitro*. Yang et al. [24] also showed that hyperoxia activated the transcription of *Rev-erb*α via the NRF2-binding site presented in the *Rev-erb*α promoter region in neonatal lung fibroblasts. Furthermore, it was found that AMPK activation destabilizes CRY proteins [25], and that HIF-1α binds to the *Per2* promoter for transactivation [26]. Thus, the potent phase shift of circadian clocks by oxidative stress is occurred through many signaling pathways.

Oxidative stress-induced phase shift of the peripheral circadian clocks might also aid in adapting to several environmental conditions, because increased ROS levels are caused by many conditions such as physiological stress, heat stress, ultraviolet radiation, and xenobiotics [7]. In our previous experiments, physiological stress and heat stress produced phase changes in the PER2::LUC rhythm of peripheral tissues [4,27]. In case of restraint stress, we identified phase advance in the peripheral PER2::LUC rhythm after 3 days of 2-h restraint stress during ZT4–6 [4]. In case of heat stress, we investigated phase advance in the peripheral PER2::LUC rhythm after warm water bath stimulation during ZT4–6 with temperature dependency [27]. Restraint stress activates glucocorticoid release as well as the sympathetic nerve system, and the administration of Dex, adrenaline, or noradrenaline caused similar phase shift in the PER2::LUC rhythm of the peripheral tissues through the glucocorticoid- or CREB-responsive elements in the promoter region of several clock genes [4,28,29]. In addition to the glucocorticoid and sympathetic nerve activation, oxidative stress might be involved in stimulus-induced phase changes of the circadian clocks.

In summary, to our knowledge, this is the first study to investigate the phenomena underlying the adaptation of the circadian clock system to oxidative stress *in vitro* and *in vivo*. Because the circadian clock also regulates the response to oxidative stress, adaptation of the clock to temporal oxidative stress may be necessary to prepare the response to other temporal oxidative stimuli as well as to maintain homeostasis.

## Competing Interests

The authors declare that they have no competing interests.
